# Simulation and Analysis of Anodized Aluminum Oxide Membrane Degradation

**DOI:** 10.3390/s23249792

**Published:** 2023-12-13

**Authors:** Saher Manzoor, Faheem Qasim, Muhammad Waseem Ashraf, Shahzadi Tayyaba, Nimra Tariq, Agustín L. Herrera-May, Enrique Delgado-Alvarado

**Affiliations:** 1 Department of Electronics, Institute of Physics, GC University Lahore, Lahore 54000, Pakistan; sahermanzoor1@gmail.com (S.M.); faheem.qasim@gcu.edu.pk (F.Q.); 2 Department of Information Sciences, Division of Science and Technology, University of Education, Township Campus, Lahore 54000, Pakistan; 3 Department of Physics and Mathematics, Faculty of Sciences, The Superior University Lahore, Lahore 54000, Pakistan; minnayjabeen@gmail.com; 4 Micro and Nanotechnology Research Center, Universidad Veracruzana, Boca del Rio 94294, Mexico; leherrera@uv.mx (A.L.H.-M.); endelgado@uv.mx (E.D.-A.)

**Keywords:** anodic aluminum oxide, membrane degradation, filtration, two-step anodization

## Abstract

Microelectromechanical systems (MEMS)-based filter with microchannels enables the removal of various microorganisms, including viruses and bacteria, from fluids. Membranes with porous channels can be used as filtration interfaces in MEMS hemofilters or mini-dialyzers. The main problems associated with the filtration process are optimization of membrane geometry and fouling. A nanoporous aluminum oxide membrane was fabricated using an optimized two-step anodization process. Computational strength modeling and analysis of the membrane with specified parameters were performed using the ANSYS structural module. A fuzzy simulation was performed for the numerical analysis of flux through the membrane. The membrane was then incorporated with the prototype for successive filtration. The fluid flux and permeation analysis of the filtration process have been studied. Scanning electron microscope (SEM) micrographs of membranes have been obtained before and after the filtration cycles. The SEM results indicate membrane fouling after multiple cycles, and thus the flux is affected. This type of fabricated membrane and setup are suitable for the separation and purification of various fluids. However, after several filtration cycles, the membrane was degraded. It requires a prolonged chemical cleaning. High-density water has been used for filtration purposes, so this MEMS-based filter can also be used as a mini-dialyzer and hemofilter in various applications for filtration. Such a demonstration also opens up a new strategy for maximizing filtration efficiency and reducing energy costs for the filtration process by using a layered membrane setup.

## 1. Introduction

Microfluidics is a branch of BioMEMS and refers to the study of fluids on a small scale. The size of MEMS devices can be measured in millimeters and micrometers. MEMS can be built with moving parts like microgears, micromotors, microvalves, and micropumps that use electrical energy to perform mechanical movements. Handheld devices can be designed with remote settings using MEMS technology. To perform absolute separation of micron-sized particles from fluids, MEMS microfilters are crucial [1,2]. In the field of novel ion transportation, nanoscale fluidic channels have gathered substantial attention. Membranes with homogenous channels can effectively separate solutes of low molecular weight from fluids [3,4]. Categorizing the membrane filtration process is performed by using a semi-permeable membrane and driving force for the flow of fluid. The driving force may be produced due to concentration, pressure, or electric potential. Overall, the filtration process is based on pressure or diffusion process. Removal of impurities from water and wastewater treatment is essential to eliminating health threats caused by environmental pollution. Many industries are producing harmful, toxic, mutagenic, and carcinogenic waste. The textile dyeing industry consumes large quantities of water during its different dyeing and finishing steps and also releases dye into natural waterways. Metal–organic frameworks have received attention for separation and adsorption applications due to their controllable pores and high porosity. But the use of metal–organic frameworks for purification and separation purposes is limited [5,6,7,8,9,10]. Concerning water treatment, assessment of membrane technology is crucial [11,12]. Due to its convenient operation, membrane separation has been increasingly used to treat inorganic wastes. There are four types of membrane filtration: microfiltration, ultrafiltration, nanofiltration, and reverse osmosis [13]. Ultrafiltration membranes have pore sizes between 2 and 100 nm. This range of pore size is suitable for removing all sorts of microbiological threats and considerable virus removal, as the size range of the viruses lies between 30 and 300 nm [11,14]. The selection of membrane material and preparation methods are the foundations of membrane morphology. Novel candidates for membrane technology should have easy and precisely controllable membrane formation with homogenous structure and stability [15].

Anodic protection has been widely used to reduce metal corrosion [16]. Anodization of aluminum substrate results in a porous membrane. Anodic aluminum oxide (AAO) has important characteristics like uniform pore structure, high aspect ratio, low fabrication cost, simplicity of fabrication with controllable geometric features, non-destructiveness, and bio-compatibility. Due to these features, it has gathered great attention for applications in biotechnology [17]. The researchers performed numerous investigations to study the effect of the anodization parameters on the morphology of AAO membranes [18,19,20,21]. According to the reported literature, AAO has been used in a wide range of applications, including drug delivery [22], catalysts [23], biosensing [24], cancer treatments [25], nanoparticle separation [26], and filtration processes [27].

Control over the geometric features of the membrane structure plays a vital role in all applications. The variation in parameters of the anodization process can affect the controlled geometry of AAO. The high aspect ratio of AAO and its self-ordered structure-property make it a suitable candidate for many nanofluidic biological/microelectromechanical systems (BioMEMS) applications [28,29,30,31]. For in vitro analysis, nanoporous membranes are an obvious choice for use. Biocompatibility and easy integration with medical implants make AAO membranes suitable candidates to be used in BioMEMS [32].

MW Ashraf et al. [33] reported the controlled pore diameter fabrication of the AAO membrane using a two-step anodization process. MW Ashraf et al. [34] investigated the suitability of the AAO membrane for dialysis by performing structural simulations. The results of this study suggest that the membrane can bear pressure up to 0.79 MPa. Poinern et al. [35] used kidney epithelial cells to test the biocompatibility of AAO membranes, and the results showed that those were an excellent substrate for growing kidney epithelial cells. Phuong, N. et al. [36] studied AAO membranes as a working template by injecting functionalized nanoparticles into the membrane. These embedded nanoparticles result in smaller voids and enhanced filtration. Patel, Y. et al. [27] reported the concept of vibro-active AAO filters in the hydromechanical system for filtration and separation. Two-layered and functionalized AAO membrane has been reported for speedy hemodialysis, early detection of cancerous cells by potential biomarkers, water purification, and exosome separation [37,38,39,40]. The significant complications associated with membrane filtration are energy consumption and fouling problems. It is essential to study the permeability and degradation of the membrane during the filtration process [41].

In this paper, a MEMS filter is developed to separate particles from suspension. An AAO membrane has been fabricated using the two-step anodization method. The low-cost fabrication method results in uniform pore arrays. The micro-fabricated membrane acts as a fluidic filter through which the sample fluid is driven for separation based on its size and morphology. Municipal fluid with a pH range between 6.5 and 8 and a density close to that of blood is used for filtration experiments. The filtration efficiency and degradation of the AAO membrane were studied using a self-designed filtration prototype. The strength analysis of AAO was performed using the ANSYS workbench. Flux was numerically analyzed using fuzzy simulations. The scanning electron microscope (SEM) characterization of the membrane has been performed before and after the filtration. An analysis of membrane permeation and flux is performed practically, and the effect of backflushing on the clogged membrane was also studied. This paper presents an extensive exploration of the degradation mechanism of AAO membranes utilized in filtration systems. What makes this research novel is its innovative blend of simulation techniques and in-depth analysis, offering a comprehensive understanding of how these membranes degrade under the influence of filtration. By employing advanced simulation models and analytical methodologies, the study unravels the intricate changes that occurred in the membrane structure, porosity, and overall integrity over prolonged filtration periods. The AAO membrane was used without any treatment for filtration. The pore size used for the filtration process is relatively small, and the permeance of the membrane is high. Also, the process is completely optimized before application using computational analysis, which further reduces the filtration cost [42].

## 2. Materials and Methods

Two-step anodization was used for the fabrication of the AAO membrane. The anodization was carried out using acetone, perchloric acid, ethanol, phosphoric acid, chromic acid, aluminum substrate, and glassware. Analytically grade 99 percent pure Sigma Aldrich aluminum was used for sampling with a thickness of 0.5 um. The diameter of the circular membrane was 4 cm. Computational analysis of membrane strength was performed using the ANSYS workbench. A static structural model was used to build membrane under actual conditions. The analysis consisted of 3D geometry, meshing, and boundary conditions to predict the strength of the filter. The numerical analysis of flux through a membrane filter was calculated using fuzzy simulation. Various variables were selected to see the flux variations. After applying rules, defuzzification was performed to calculate the crisp value of the output. Mamdani’s model was used to check the accuracy of the obtained result. The MEMS-based filter with an integrated AAO membrane was used to perform filtration practically.

## 3. Fabrication

Fabrication consists of three stages. The first stage is the pre-treatment of the substrate. In this stage, the aluminum substrate and all the glassware were sonicated in the presence of acetone to remove all the impurities. In order to obtain a smooth surface for the anodization process, the substrate was electropolished at 5 °C and 20 V using 1:4 perchloric acid and ethanol.

**Figure 1:**
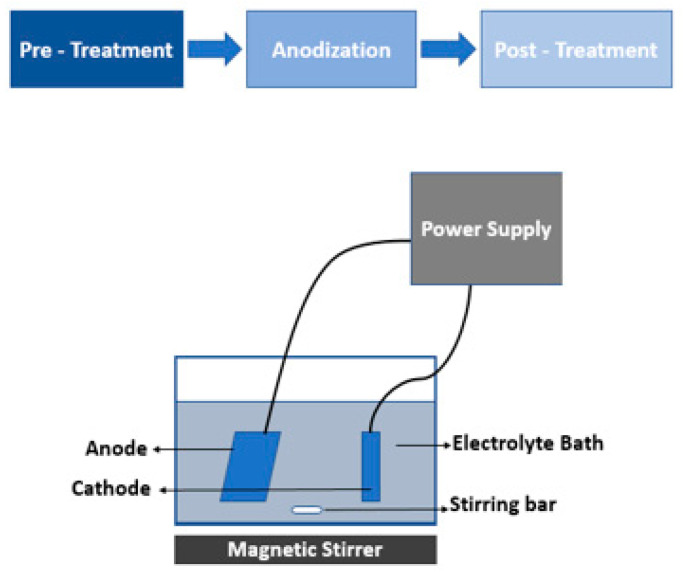
Schematic of the anodization process for the fabrication of the AAO membrane.

The setup for anodization is shown in Figure 1. The aluminum substrate was used as an anode in the electrolyte bath of 0.5 M oxalic acid in the presence of lead as the cathode. The anodization set up was maintained at 0 degrees under constant stirring. In the second stage, a two-step anodization was performed. First, mild anodization was performed at the substrate with a starting voltage of 45 V that was gradually increased to 125 V. After the voltage of 125 V was achieved, the step was carried out for 28 min. Then, hard anodization was performed for 190 min at a voltage of 125 V. The substrate was etched using 5% phosphoric acid for about 30 min between the two steps.

After the anodization, the anodized membrane was post-treated. In this stage, the membrane was etched in the presence of acid. Once the anodization procedure was completed, the barrier layer was removed using a negative polarity voltage in 0.4 M potassium chloride.

The membrane fabrication process was optimized. The porous membrane can be regenerated using the mentioned anodization conditions.

## 4. Experimental Design for Filtration

**Figure 2:**
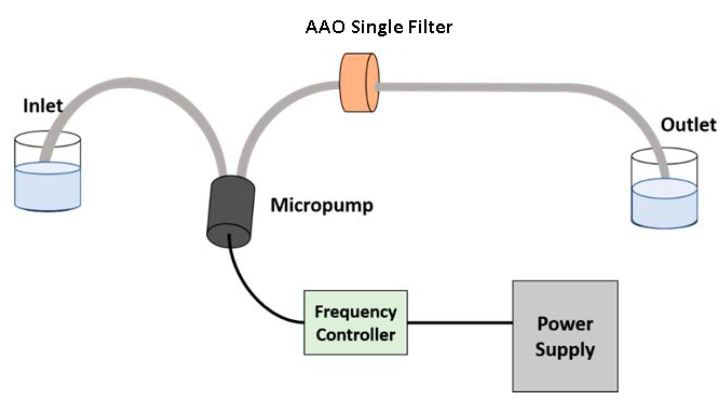
Schematic diagram design for practical filtration.

The membrane filtration setup comprises components like a micropump, a frequency controller, a porous AAO membrane, and fluid samples. This experimental setup was used with a single AAO filter by Saher [42] and Faheem et al. [43] used the same experimental setup with two stages of an AAO filter in the filtration process. This experimental setup was developed by Saher Mansoor and Faheem Qasim collectively. In both studies, the fluid samples were taken from the same area, Ichhra, Lahore, Pakistan, that contained the same kinds of contamination. A micropump plays a vital role in membrane filtration. The micropump in membrane filtration is responsible for manipulating and controlling the delivery of miniature fluids. The pump has been connected toa frequency controller to rectify the fluid flow. The AAO membrane acts as a barrier that restricts the transport of unwanted material in a selective manner. Other components are pipes for fluid flow from the inlet towards the outlet and a power supply for providing the necessary mechanical energy for membrane actuation. The designed setup for filtration, along with its schematic, is shown in Figure 2.

For practical filtration using the AAO membrane, a certain volume of water was taken into the feed beaker, where the inlet pipe was immersed. The apparatus then operates at constant pressure, and the solution permeates through the AAO membrane, leaving behind the waste materials. The permeated material is then collected from the outlet in the permeate beaker.

## 5. Results and Discussion

### 5.1. Computational Analysis

During the filtration process, the fluid exerts a certain amount of force on the filtration membrane, so strength analysis of the membrane plays an important role. The strength of the porous membrane is different from the strength of the nonporous membrane. The ANSYS static structural module can be used for strength prediction of the fabricated membrane with specified parameters. To drag the fluid across the membrane during the filtration process, the pressure gradient ranges from 50 to 350 kPa. The membrane must be strong enough to bear pressure up to this range to function properly for application.

**Figure 3:**
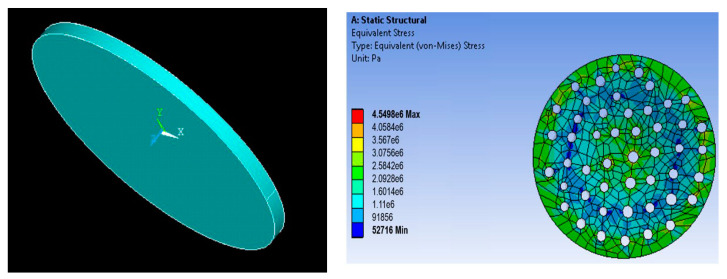
3D geometry and stress distribution in the membrane.

**Figure 4:**
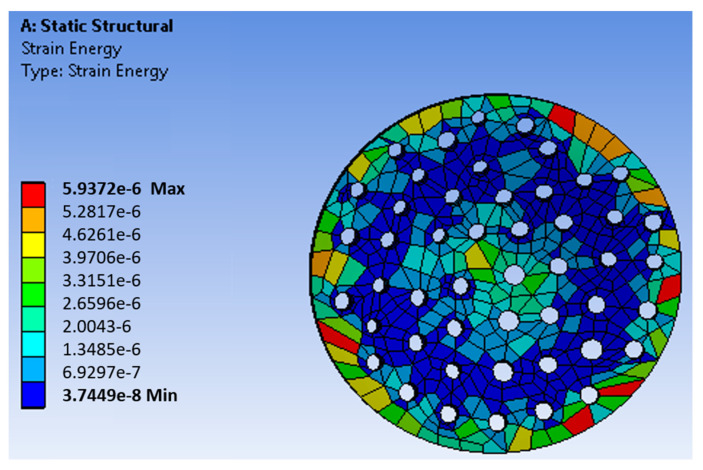
Strain distribution in an AAO membrane filter.

**Figure 5:**
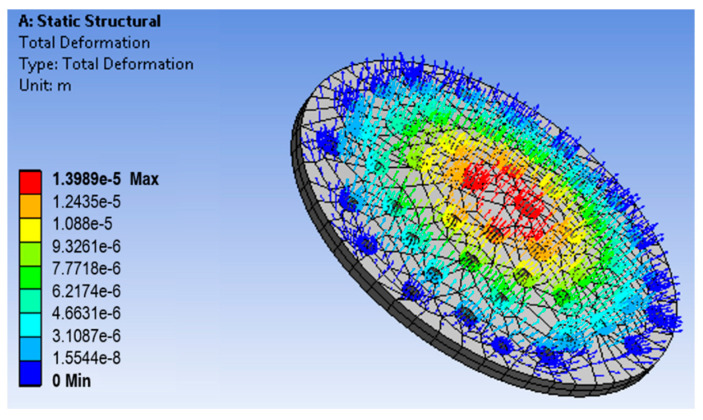
Mesh model uniform vector field display for total deformation analysis of the membrane filter.

**Figure 6:**
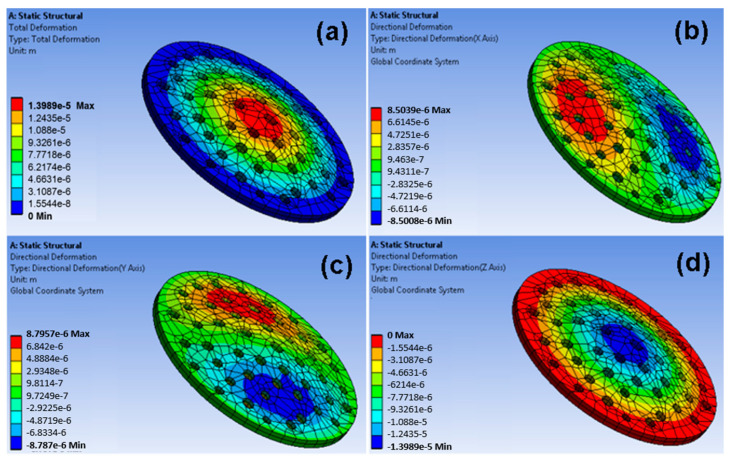
Deformation analysis of the membrane filter: (**a**) total deformation, (**b**) directional deformation along the X-axis, (**c**) directional deformation along the Y-axis, and (**d**) directional deformation along the Z-axis.

A porous membrane has been designed on the workbench using the parameters of the fabricated membrane. The membrane was then meshed, and boundary conditions were applied. The fixed support for the membrane was set, and the driving force of 350 kPa was applied at the top of the membrane. The fluid flow through the filter produced the stress in the filter that caused strain in the mechanical structure. Figure 3 shows the directions of the axes X, Y, and Z of the membrane and the uniform stress distribution. The Z-axis denotes the thickness. Figure 4 shows the strain distribution on the filter membrane. It has been observed that deflection occurs at the center of the membrane along the negative Z-direction. Figure 5 represents the uniform vector field for membrane deformation, and Figure 6a represents the total directional deformation of the membrane along all three dimensions. Figure 6b,c represent the directional deformation in the X and Y directions. The magnitude of X and Y directional deformation is different due to the variation in the pore distribution on either side. Figure 6d shows the Z-axes deformation. The stress and strain do not exceed the yield strength of the porous membrane, and the results indicate that the membrane filter can bear pressure up to 300 kPa.

### 5.2. Numerical Analysis

**Figure 7:**
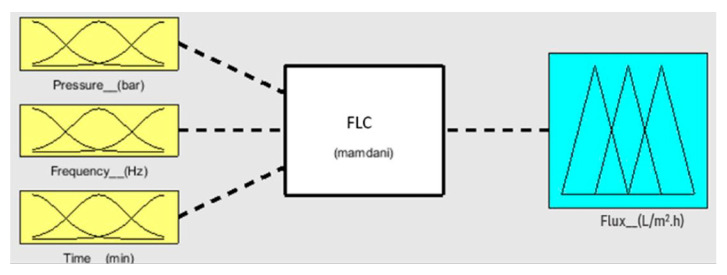
FLC interface with inputs versus output.

**Figure 8:**
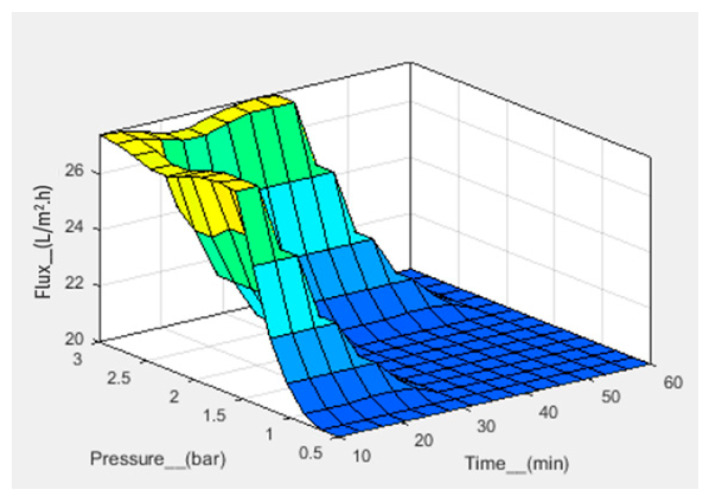
3D graph for flux with pressure and time as input.

**Figure 9:**
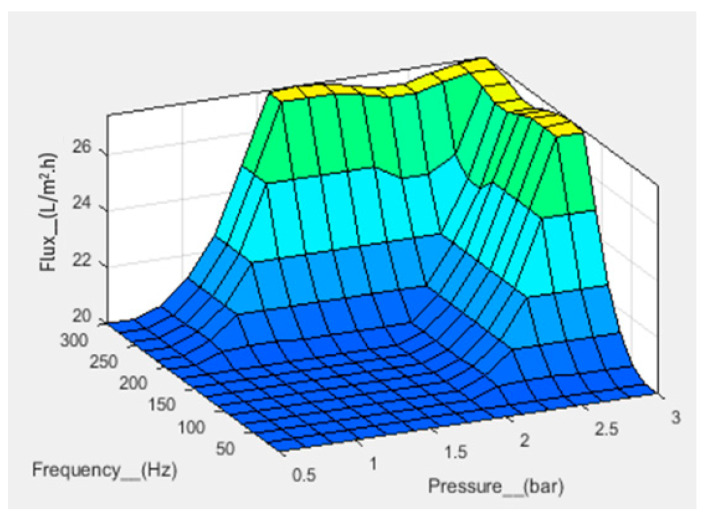
3D graph for flux with frequency and pressure as input.

**Figure 10:**
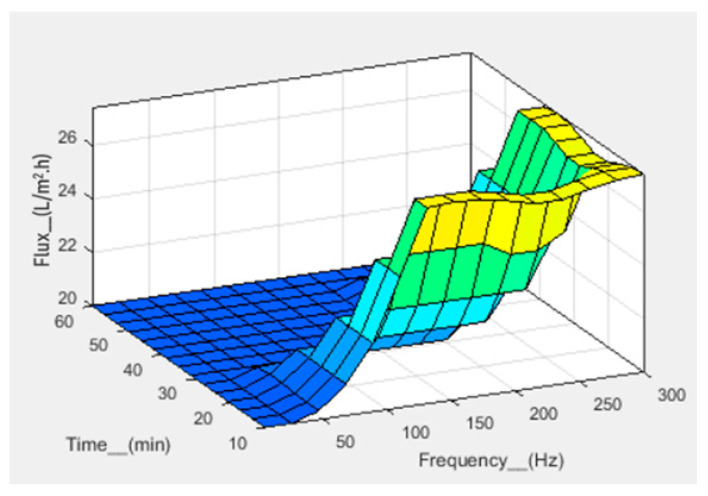
3D graph for flux with time and frequency as input.

**Figure 11:**
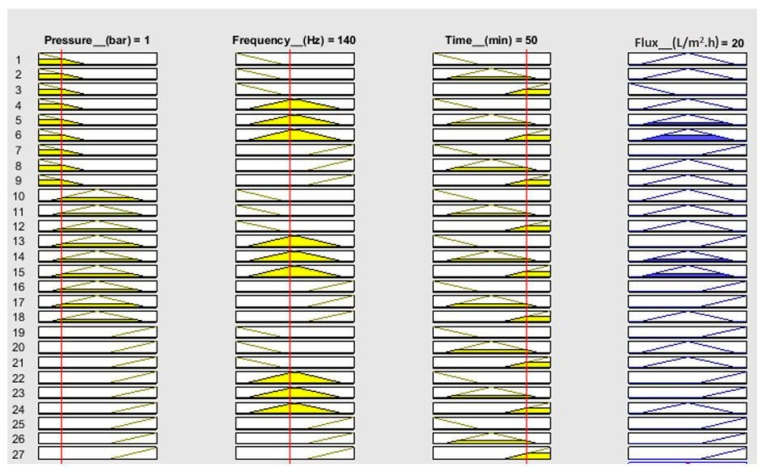
Rule viewer graph for crisp values.

Numerical analysis for membrane flux was performed using fuzzy simulations. Fuzzy analysis provides support for predicting output using theoretical data before practical application. Fuzzification was performed in a fuzzy simulation using three inputs against one output. For simulation, pressure, frequency, and time have been taken in bar, hertz, and minutes, respectively, with corresponding output flux in L/m^2^.h. The fuzzy logic controller (FLC) interface is shown in Figure 7. For simulation, the range of all inputs was defined, and three membership functions (Mf) were assigned to each input. The range of all defined membership functions is given in Table 1. The triangular Mf type was used due to its simple implementation and fast computation. A total of 27 rules have been defined. The simulation provides three-dimensional graphs between inputs and output, as shown in Figure 8, Figure 9 and Figure 10. From the rule viewer graph shown in Figure 11, crisp values of all the inputs are noted to calculate and verify results using Mamdani’s model.

For calculations of flux, crisp values from the rule viewer graph are used in Mamdani’s model. The calculated Mfs for input 1 (pressure) are calculated as k1 = 0.67 and k2 = 0.33. Similarly, Mfs for input 2 (frequency) are k3 = 0.53 and k4 = 0.47. For input 3 (time), the Mfs are calculated as k5 = 0.17 and k6 = 0.83.

The results are tabulated by selecting 8rules out of 27 rules for Mf k1, k2, k3, k4, k5, and k6 values. The calculations are given in Table 2, where the “^” sign represents the comparison between Mf and the minimum value.

Figure 10 shows that at a pressure of 1 bar, a frequency of 140 Hz, and a time of 50 min, the flux value is 20 L/m^2^.h. The calculated value of flux, that is, 20.7 L/m^2^.h, is in agreement with the simulated value.

### 5.3. Membrane Morphology

**Figure 12:**
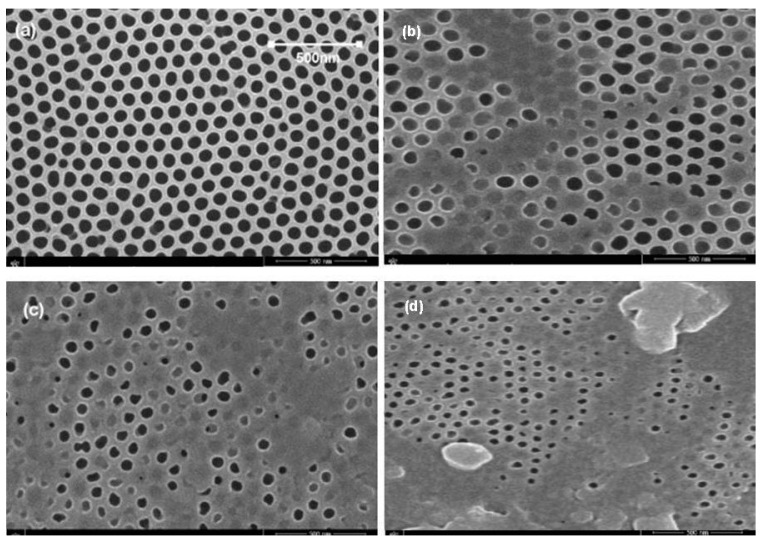
SEM micrographs: (**a**) fabricated AAO membrane, (**b**) membrane after 2 cycles of filtration, (**c**) membrane after 4 cycles of filtration, and (**d**) membrane after 6 cycles of filtration.

The SEM is used for the characterization of the fabricated AAO membrane. The SEM micrographs are shown in Figure 12. Figure 12a shows the fabricated membrane. In contrast, Figure 12b–d represents the membrane after 2, 4, and 6 cycles of filtration, respectively. SEM reveals that the fabricated membrane has an average pore size of 80 nm. After filtration, the clogging of the pores can be seen clearly. But it was observed that the membrane was mechanically stable.

### 5.4. Permeation Analysis

In the membrane filtration process, unwanted materials can be removed from water by adsorption or repulsion mechanisms. In fouling and flux analysis, adsorbed solutes on the membrane surface and desorbed fluid through the membrane are important. Pure water flux “Φ” is calculated as the total volume of permeated water “V” divided by the product of effective membrane area “A” and time of operation “t”, as given in the following equation:

φ = V/At(1)

The permeability of a porous membrane depends on pore size, the properties of the fluid, and the driving force. The permeation of a porous AAO membrane is calculated by Darcy’s law given in Equation (2):

μ = QηL/A∆P(2)

where Q is the flow rate, η is the viscosity of the fluid, L is the length, A is the effective area, and ∆P is the pressure change.

The geometric features of the fabricated membrane and filtration parameters are given in Table 3.

### 5.5. Membrane Degradation Analysis

In membrane degradation analysis, various mechanisms can occur during filtration. The membrane can be clogged intermediately due to filter condition. The chemical dissolution of membrane material can also occur. The chemically dissolute membrane material is then deposited into the pores, which results in a clogged pore. In this study, the pH of the solution was not high enough, so membrane fouling mostly occurred due to the deposition of unwanted materials on the membrane during filtration. After six cycles, the clogging was very high; this may be due to the re-deposition of dissolute water-soluble complexes.

**Figure 13:**
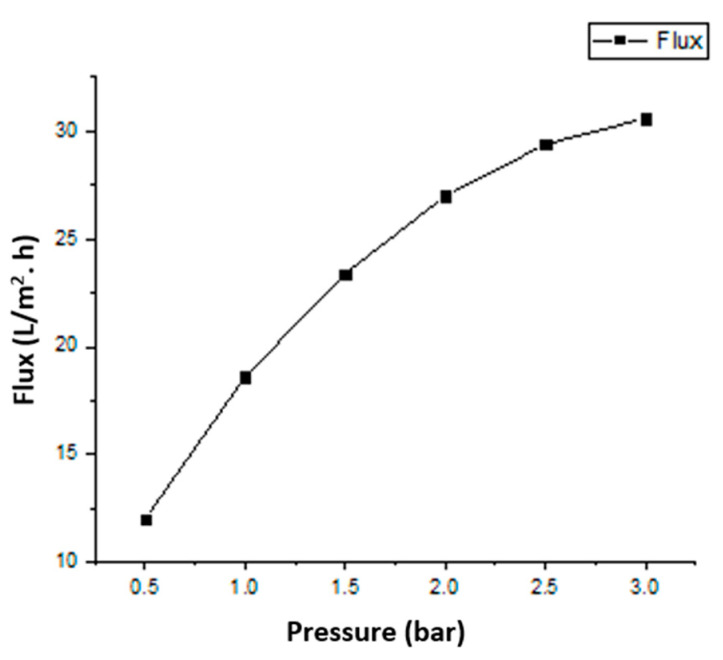
Flux variation at different pressure values.

**Figure 14:**
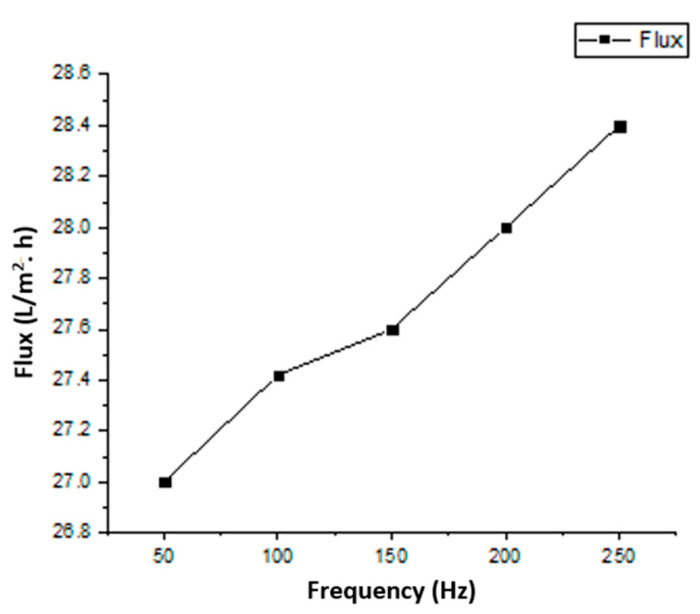
Flux variation at different frequencies.

**Figure 15:**
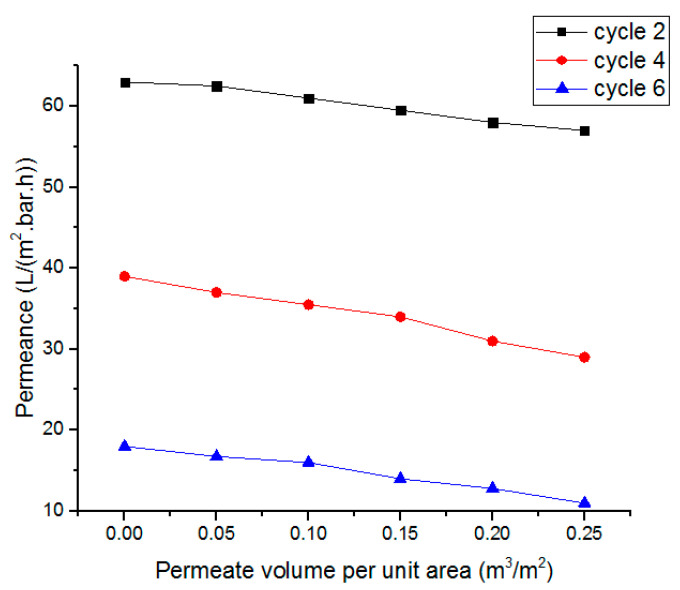
Variation of permeance with specified permeate volume per unit area.

The liquid flux of the AAO membrane linearly depends on the pressure, which shows agreement with Darcy’s law. Figure 13 and Figure 14 represent the dependence of fluid flux on pressure and frequency, respectively. The clogging of membrane pores resulted in a rapid flux decrease. In the first cycle of fluid flow, the clogging did not significantly affect the flux rate, but after six cycles, the clogging of pores increased and the flux rate decreased rapidly. The dependence of the specified permeate volume on permeance is shown in Figure 15. In biological terms, the stable membrane refers to the membrane that does not change characteristic properties during application under certain constraints. The stability of the fabricated membrane was first checked with an ANSYS simulation. During the experimental filtration, the membrane did not rupture and was also stable to buckling. It indicated that the membrane was durable under the defined conditions.

Through fuzzy simulation and practical results, it was clear that the frequency slightly affected fluid flux, whereas pressure strongly affected the flux rate. The fluid flow during filtration was in agreement with Poiseuille’s equation. After six cycles, the membrane pores were highly clogged and affected the membrane’s permeance. The membrane, after filtration, was then backflushed with DI water and sonicated to remove impurities. The backflushed membrane after two cycles of filtration showed a permeance value close to the permeance of the initially fabricated membrane. It showed that the effect of water-soluble complexes was not significant. After multiple filtration cycles, the membrane degrades chemically due to water-soluble complexes, a thick layer of deposited foulant on the surface, and wholly occluded pores with foulants. When the solute particles are smaller than the pore diameter, the membrane pores are clogged from inside. The particles larger than the pore diameter are deposited on the surface of the membrane. The internal and surface-deposited particles led to a reduction in pore size that resisted fluid flow. Figure 12d shows that the uniform pore size of the fabricated membrane is significantly reduced, and the pores are clogged due to deposited foulant. After multiple cycles, the membrane requires chemical cleaning to restore flux and permeation. However, it was observed that, after two cycles, the backflushing effect was significant. Thus, the fabricated membrane did not initially degrade due to chemicals and showed better permeation results. This membrane and the designed prototype are suitable for separation and filtration applications. The advantages of fabricated membranes include a low-cost fabrication process, easy maintenance, and a small-sized filtration prototype. Due to the small size of the membrane pores, a wide range of contaminants can be removed from the solvent, including viruses. The disadvantage is that the AAO membrane is brittle and can break if it is not handled with care. The fouling of the membrane also occurs after the filtration process. However, certain procedures, like backflushing and chemical treatments, may be used to reduce the fouling effect. This setup can be used as BioMEMS for hemofiltration with a few modifications. The MEMS filter can also be used in MEMS-based handheld devices for hemofiltration and artificial kidneys.

## 6. Conclusions

A nanoporous membrane with a pore size of around 80 nm was fabricated successfully and applied for the filtration of municipal water. The fabricated AAO membrane, with its high porosity and uniform pore size of ~80 nm, enables successive removal of unwanted materials in the BioMEMS-based filtration prototype. The filter membrane’s strength is crucial in the filtration process. Computational analysis was performed using a static structural workbench to study membrane strength, as prolonged and continuous fluid flow might reduce the filter strength. The analysis shows that the filter membrane can bear a pressure of 300 kPa. Before the practical application of the membrane incorporated into the filtration setup, flux through the membrane was calculated using fuzzy simulation. Fouling of the membrane occurs due to the increased thickness of the deposited material on the membrane, and with the passage of time, the pore size is reduced. After multiple cycles, the pores become fully clogged. The permeance of the membrane is high as compared to earlier reports. After two cycles, the pores are not chemically affected due to water-soluble complexes, so the membrane can be reused after backflushing. After multiple filtration cycles, the effect of water-soluble complexes is significant, and only backflushing cannot retain the filtration capability of the membrane. The numerically analyzed results using fuzzy simulation were in agreement with the practical results. This membrane, with its designed prototype, can be effectively used for filtration and separation applications in biotechnology. The model can also be applied as a BioMEMS for plasma filtration and separation of various nanosized, unwanted materials from fluids. MEMS is enabling the technology and application of porous membranes in blood and water filtration. The MEMS-based filtration setup can be applied as a mini-dialyzer or hemofilter. If more than one membrane filter is incorporated into the setup with varied pore sizes, this filtration setup can also obtain maximum membrane efficiency for filtration with reduced energy consumption.

## Figures and Tables

**Table 1:** Range of membership functions with their corresponding inputs and outputs.

No.	Pressure (bar)		Frequency (Hz)		Time (min)		Flux (L/m^2^.h)	
Mf1	Low	0.5~1.5	Low	5~120	Low	10~30	Low	10~18
Mf2	Medium	0.75~2.75	Medium	35~270	Medium	15~55	Medium	12~28
Mf3	High	2~3	High	185~300	High	40~60	High	22~30

**Table 2:** Calculations of flux.

Rule no.	Pressure (bar)	Frequency (Hz)	Time (min)	Flux (L/m^2^.h)	Comparison between Membership Function Value	Min. Value (Mi)	Singleton Value for Flux (Si)	Mi × Si
R1	Low	Low	Low	Medium	k1^k3^k5	0.17	0.2	0.034
R2	Low	Low	Medium	Medium	k1^k3^k6	0.53	0.2	0.106
R3	Low	Low	High	Low	k1^k4^k5	0.17	0.1	0.017
R4	Medium	Medium	Medium	Medium	k1^k4^k6	0.47	0.2	0.094
R5	Medium	Medium	High	Medium	k2^k3^k5	0.17	0.2	0.034
R6	Medium	High	High	Medium	k2^k3^k6	0.33	0.2	0.066
R7	High	Medium	High	Medium	k2^k4^k5	0.17	0.2	0.034
R8	High	High	High	High	k2^k4^k6	0.33	0.3	0.099

Σ Mi = 2.34, Σ (Mi × Si) = 0.484. Expression of Mamdani’s model = [Σ (Mi × Si)/ΣMi] × 100. The calculated value of flux = 20.7 L/m^2^.h. The simulated MATLAB value of flux = 20 L/m^2^.h. The difference between calculated and simulated values = 0.7.

**Table 3:** Features of the AAO membrane and filtration parameters.

Features of AAO Membrane		Filtration Parameters	
Pore diameter	80 nm	Pressure/driving force	1–3 bar
Interpore distance	105 nm	Viscosity	0.89 mPa.s
Porosity	52%	Density	940 kg/m^3^
Thickness	100 µm	Initial permeance	63 L/m^2^.bar.h

## Data Availability

Most of the steps and details have been provided in the manuscript. However, more detail and information can be obtained from the authors.
